# Supplementary material for: Physiology of Saccharomyces cerevisiae during growth on industrial sugar cane molasses can be reproduced in a tailor-made defined synthetic medium
Source: Sci Rep. 2023 Jun 29;13:10567. doi: 10.1038/s41598-023-37618-8 (PMC10310838; doi:10.1038/s41598-023-37618-8)
Supplement: Supplementary file 1 — Supplementary Information. [file 41598_2023_37618_MOESM1_ESM.docx]

**Physiology of *Saccharomyces cerevisiae* during growth on industrial sugarcane molasses can be reproduced in a tailor-made defined synthetic medium**

Kevy Pontes Eliodório^1, ‡^; Gabriel Caetano de Gois e Cunha^1, ‡^; Felipe Senne de Oliveira Lino^2^; Morten Otto Alexander Sommer^3^; Andreas Karoly Gombert^4^; Reinaldo Giudici^1^; Thiago Olitta Basso^1,*^

^1^Department of Chemical Engineering, Escola Politécnica, Universidade de São Paulo, Av. Prof. Luciano Gualberto, 380, 05508-010, São Paulo, Brazil

^2^ Nosh.bio GmbH, Schwarzschildstraβe 6, 12489 Berlin, Germany

^3^Novo Nordisk Foundation Center for Biosustainability, Technical University of Denmark, 2800, Kongens Lyngby, Denmark

^4^School of Food Engineering, University of Campinas, R. Monteiro Lobato 80, 13083-862, Campinas, Brazil

* Corresponding author

Email address: thiagobasso@usp.br (Basso, T.O.)

Postal address: Av. Professor Lineu Prestes 580, 05508-000, São Paulo - SP, Brazil

Telephone: + 55 11 30912260

^‡^both authors contributed equally to this work

**Supplementary data**





Figure S1 - Performance of *S. cerevisiae* Ethanol Red® in the synthetic molasses (Lino, Basso, and Sommer, 2018), in three industrial sugarcane molasses media, and in YP-sucrose (1% Yeast extract, 2% Peptone, and 18% Sucrose) in a scaled-down sugarcane biorefinery throughout five consecutive fermentation cycles [11]. The fermentations were performed in triplicates. Navy (Lino’s synthetic molasses); Red (Mol_A); Green (Mol_B); Purple (Mol_C); Light yellow (YP-sucrose). (A) Ethanol yield (as a percentage of theoretical maximum 0.511 g of ethanol.g of TRS^-1^, %) (B) Glycerol concentrations (g.L^-1^). (C) Biomass variation (%) is represented as the percentage of the first cycle initial wet yeast mass (accumulated variation). (D) Biomass variation (%) represented as percentage of the cycle initial wet yeast mass (cycle variation).





Figure S2 – Total reducing sugar content (%) of five sugarcane molasses samples (Mol_A, Mol_B, Mol_C, Mol_D, and Mol_E) and the semi-defined synthetic molasses proposed by Lino et al. [3]

Figure S3 - Growth kinetics in molasses (black triangles) and modified versions of 1SMol with variations in ammonium and vitamin content (colored lines) for S. cerevisiae strains PE-2 and CEN.PK-113.7D

Table S1 - Compositional variations concerning 2SMolAA25 used in microplate assays to assess effect on yeast growth parameters with coded values in brackets.

| Condition | Concentration ratio of 2SMol | |
| --- | --- | --- |
|  | Lower | Upper |
| 2SMolAA25 | - | |
| Inorganic nitrogen | 0 (-1) | 2 (+1) |
| Organic Acids | 0 (-1) | 2 (+1) |
| Trace elements | 0.1 (-1) | 10 (+1) |
| Vitamins | 0.1 (-1) | 2 (+1) |
| Mg & K | 0.2 (-1) | 2 (+1) |
| Calcium | 0 (-1) | 2 (+1) |
| Organic nitrogen | 0 (-1) | 0.5 (+1) |
| Phosphate | 0.5 (-1) | 2 (+1) |

*The tested conditions are presented as the ratio between theses variations for each nutritional group and the composition of 2SMol containing 25% of amino acids (2SMolAA25)


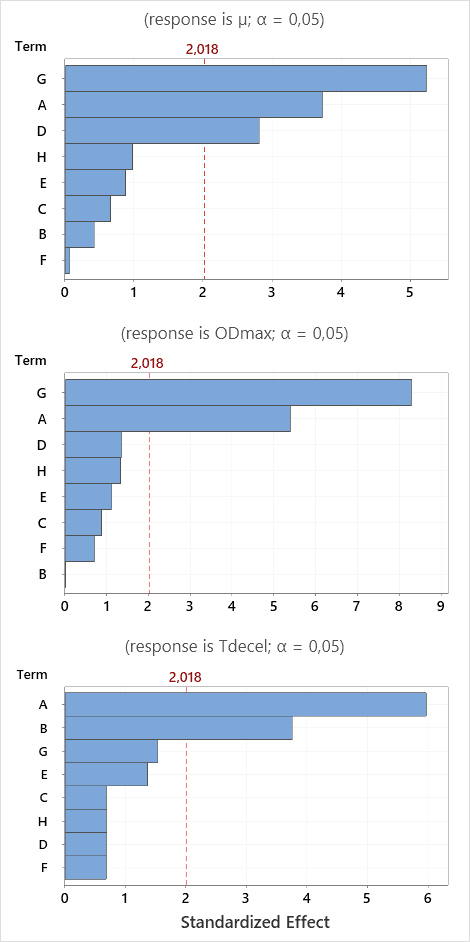

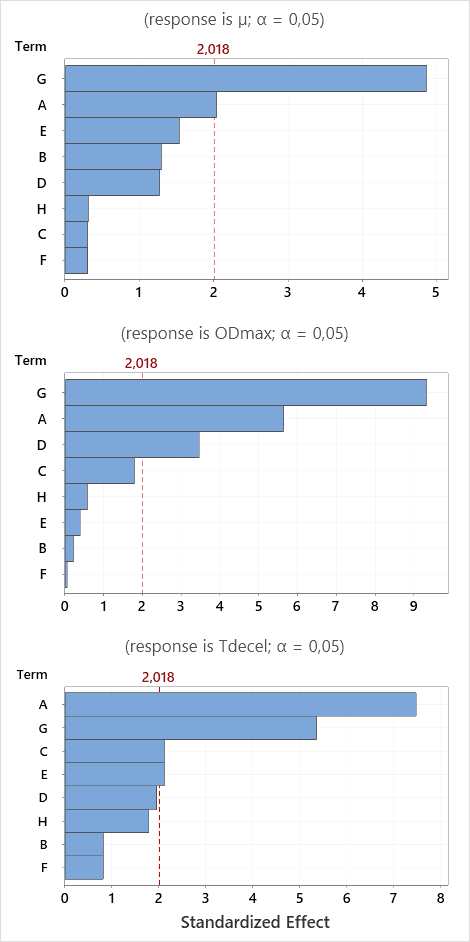


PE-2

CEN.PK113-7D

Figure S4 - Pareto chart of the standardized effects for CEN.PK113-7D and PE-2 in 2SMolAA25 with variations in the concentration of nutritional groups. (A) Inorganic nitrogen, (B) Organic acids, (C) Trace elements, (D) Vitamins, (E) Mg and K, (F) Calcium, (G) Organic nitrogen, and (H) Phosphate.

Table S2 - Growth parameters for *S. cerevisiae* strains PE-2 and CEN.PK113-7D using lower and higher levels of eight nutritional groups of 2SmolAA25.

| **PE-2** | | | | | | |
| --- | --- | --- | --- | --- | --- | --- |
| Parameter | µ (h^-1^) | | OD_Max_ | | T_decel_ (h) | |
| Level | Lower | Higher | Lower | Higher | Lower | Higher |
| **2SMolAA25** | 0.416 | | 0.823 | | 12.22 | |
| **Inorganic nitrogen** | 0.428 | 0.4 | 0.812 | 0.688 | 8.22 | 13.33 |
| **Organic Acids** | 0.432 | 0.414 | 0.796 | 0.8 | 10.67 | 10.11 |
| **Trace elements** | 0.407 | 0.403 | 0.778 | 0.817 | 11.78 | 10.34 |
| **Vitamins** | 0.393 | 0.411 | 0.736 | 0.812 | 13 | 11.67 |
| **Mg & K** | 0.433 | 0.412 | 0.789 | 0.798 | 12 | 10.56 |
| **Calcium** | 0.425 | 0.421 | 0.81 | 0.809 | 12.22 | 11.67 |
| **Organic nitrogen** | 0.357 | 0.424 | 0.682 | 0.889 | 13.33 | 9.67 |
| **Phosphate** | 0.419 | 0.415 | 0.787 | 0.8 | 11.56 | 12.78 |
| Mol_B | 0.496 | | 0.902 | | 5.78 | |
| Mol-A | 0.426 | | 0.682 | | 8.56 | |
| Mol_D | 0.417 | | 0.746 | | 17.56 | |
| **CEN.PK113-7D** | | | | | | |
| Parameter | µ (h^-1^) | | OD_Max_ | | T_decel_ (h) | |
| Level | Lower | Higher | Lower | Higher | Lower | Higher |
| **2SMolAA25** | 0.387 | | 0.833 | | 17 | |
| **Inorganic nitrogen** | 0.404 | 0.367 | 0.857 | 0.689 | 13.11 | 17 |
| **Organic Acids** | 0.382 | 0.387 | 0.813 | 0.812 | 14.22 | 16.67 |
| **Trace elements** | 0.367 | 0.373 | 0.852 | 0.825 | 16.11 | 16.56 |
| **Vitamins** | 0.348 | 0.377 | 0.836 | 0.878 | 16.89 | 16.45 |
| **Mg & K** | 0.388 | 0.379 | 0.84 | 0.806 | 16.89 | 16 |
| **Calcium** | 0.384 | 0.385 | 0.818 | 0.84 | 16.89 | 16.45 |
| **Organic nitrogen** | 0.335 | 0.388 | 0.638 | 0.896 | 17 | 16 |
| **Phosphate** | 0.365 | 0.375 | 0.797 | 0.838 | 16.22 | 16.67 |
| Mol_B | 0.390 | | 0.827 | | 10.78 | |
| Mol_A | 0.380 | | 0.666 | | 14.56 | |
| Mol_D | 0.370 | | 0.716 | | 26 | |

* Standard deviations are not shown, given that they represented less than 8% of each mean value.

Table S3 - Investigated factors for the 3³ factorial design and their levels with coded values in brackets.

| Investigated factors | Factor levels in % of 2SMol content | | |
| --- | --- | --- | --- |
| Organic nitrogen | 0 (-1) | 37,5 (0) | 75 (+1) |
| Inorganic nitrogen | 0 (-1) | 100 (0) | 200 (+1) |
| Vitamin | 0 (-1) | 100 (0) | 200 (+1) |

Table S4 - Model coefficients for maximum specific growth rate (µ) obtained from the 3^3^ factorial design for yeast strains PE-2 and CEN.PK113-7D. (1) Organic nitrogen, (2) Inorganic nitrogen, (3) Vitamins.

| Term | PE-2 | | | | CEN.PK113-7D | | | |
| --- | --- | --- | --- | --- | --- | --- | --- | --- |
|  | Coefficient | Standard Error | t-Value | p-Value | Coefficient | Standard Error | t-Value | p-Value |
| b_0_ | 0.424 | 0.006 | 68.820 | 0.000 | 0.401 | 0.008 | 53.100 | 0.000 |
| b_1_ | 0.044 | 0.003 | 15.540 | 0.000 | 0.046 | 0.004 | 13.030 | 0.000 |
| b_2_ | 0.026 | 0.003 | 9.130 | 0.000 | 0.032 | 0.004 | 9.130 | 0.000 |
| b_3_ | 0.023 | 0.003 | 8.230 | 0.000 | 0.035 | 0.004 | 9.890 | 0.000 |
| b_11_ | -0.037 | 0.005 | -7.550 | 0.000 | -0.048 | 0.006 | -7.860 | 0.000 |
| b_22_ | -0.023 | 0.005 | -4.680 | 0.000 | -0.033 | 0.006 | -5.460 | 0.000 |
| b_33_ | -0.024 | 0.005 | -4.810 | 0.000 | -0.031 | 0.006 | -5.170 | 0.000 |
| b_12_ | -0.030 | 0.003 | -8.510 | 0.000 | -0.048 | 0.004 | -11.140 | 0.000 |
| b_13_ | 0.006 | 0.003 | 1.730 | 0.089 | 0.008 | 0.004 | 1.870 | 0.065 |
| b_23_ | 0.011 | 0.003 | 3.010 | 0.004 | 0.006 | 0.004 | 1.330 | 0.188 |
| R^2^ | 89.08% | | | | 89.40% | | | |
| R^2^ adjusted | 87.69% | | | | 88.05% | | | |
| R^2^ predicted | 85.57% | | | | 86.34% | | | |

Table S5 - Model coefficients for maximum absorbance (OD_max_) obtained from the 3^3^ factorial design for yeast strains PE-2 and CEN.PK113-7D. (1) Organic nitrogen, (2) Inorganic nitrogen, (3) Vitamins.

| Term | PE-2 | | | | CEN.PK113-7D | | | |
| --- | --- | --- | --- | --- | --- | --- | --- | --- |
|  | Coefficient | Standard Error | t-Value | p-Value | Coefficient | Standard Error | t-Value | p-Value |
| b_0_ | 0.914 | 0.017 | 55.310 | 0.000 | 0.903 | 0.021 | 44.040 | 0.000 |
| b_1_ | 0.159 | 0.008 | 20.840 | 0.000 | 0.167 | 0.009 | 17.580 | 0.000 |
| b_2_ | 0.076 | 0.008 | 9.890 | 0.000 | 0.074 | 0.009 | 7.840 | 0.000 |
| b_3_ | 0.032 | 0.008 | 4.230 | 0.000 | 0.044 | 0.009 | 4.650 | 0.000 |
| b_11_ | -0.081 | 0.013 | -6.100 | 0.000 | -0.056 | 0.016 | -3.400 | 0.001 |
| b_22_ | -0.042 | 0.013 | -3.160 | 0.002 | -0.055 | 0.016 | -3.370 | 0.001 |
| b_33_ | -0.017 | 0.013 | -1.280 | 0.205 | -0.006 | 0.016 | -0.360 | 0.722 |
| b_12_ | -0.066 | 0.009 | -7.060 | 0.000 | -0.103 | 0.012 | -8.840 | 0.000 |
| b_13_ | 0.034 | 0.009 | 3.640 | 0.001 | 0.029 | 0.012 | 2.480 | 0.016 |
| b_23_ | 0.014 | 0.009 | 1.450 | 0.150 | 0.016 | 0.012 | 1.370 | 0.176 |
| R^2^ | 90.34% | | | | 82.35% | | | |
| R^2^ adjusted | 89.11% | | | | 82.37% | | | |
| R^2^ predicted | 87.49% | | | | 79.69% | | | |

Table S6 - Model coefficients for deceleration time (t_decel_) obtained from the 3^3^ factorial design for yeast strains PE-2 and CEN.PK113-7D. (1) Organic nitrogen, (2) Inorganic nitrogen, (3) Vitamins.

| Term | PE-2 | | | | CEN.PK113-7D | | | |
| --- | --- | --- | --- | --- | --- | --- | --- | --- |
|  | Coefficient | Standard Error | t-Value | p-Value | Coefficient | Standard Error | t-Value | p-Value |
| b_0_ | 11.137 | 0.378 | 29.480 | 0.000 | 18.274 | 0.949 | 19.260 | 0.000 |
| b_1_ | -4.315 | 0.175 | -24.670 | 0.000 | -4.889 | 0.439 | -11.130 | 0.000 |
| b_2_ | -2.297 | 0.175 | -13.130 | 0.000 | -3.704 | 0.439 | -8.430 | 0.000 |
| b_3_ | -0.766 | 0.175 | -4.380 | 0.000 | -4.463 | 0.439 | -10.160 | 0.000 |
| b_11_ | 0.352 | 0.303 | 1.160 | 0.249 | 1.148 | 0.761 | 1.510 | 0.136 |
| b_22_ | 0.037 | 0.303 | 0.120 | 0.903 | -0.444 | 0.761 | -0.580 | 0.561 |
| b_33_ | 0.704 | 0.303 | 2.320 | 0.023 | 2.871 | 0.761 | 3.770 | 0.000 |
| b_12_ | 0.111 | 0.214 | 0.520 | 0.606 | 1.482 | 0.538 | 2.750 | 0.007 |
| b_13_ | -0.620 | 0.214 | -2.900 | 0.005 | -4.084 | 0.538 | -7.590 | 0.000 |
| b_23_ | -0.796 | 0.214 | -3.720 | 0.000 | -0.806 | 0.538 | -1.500 | 0.139 |
| R^2^ | 92.13% | | | | 87.59% | | | |
| R^2^ adjusted | 91.12% | | | | 86.02% | | | |
| R^2^ predicted | 89.60% | | | | 84.04% | | | |





Figure S5 - Growth kinetics of *S. cerevisiae* strains CEN.PK 113-7D and PE-2 in: molasses (black lines, Mol_B solid, and Mol_D dashed) and modified versions of 2SMol [% of Organic nitrogen, Inorganic Nitrogen, vitamins in 2SMol]: [37.5, 100, 100] red line, [37.5, 200, 200] dark green, [37.5, 0, 200] light blue, [75, 100, 100] marine blue, [0, 200, 100] grey, [37.5, 100, 200] green, [37.5, 200, 100] light blue, [0, 100, 100] yellow, [0, 100, 200] pink


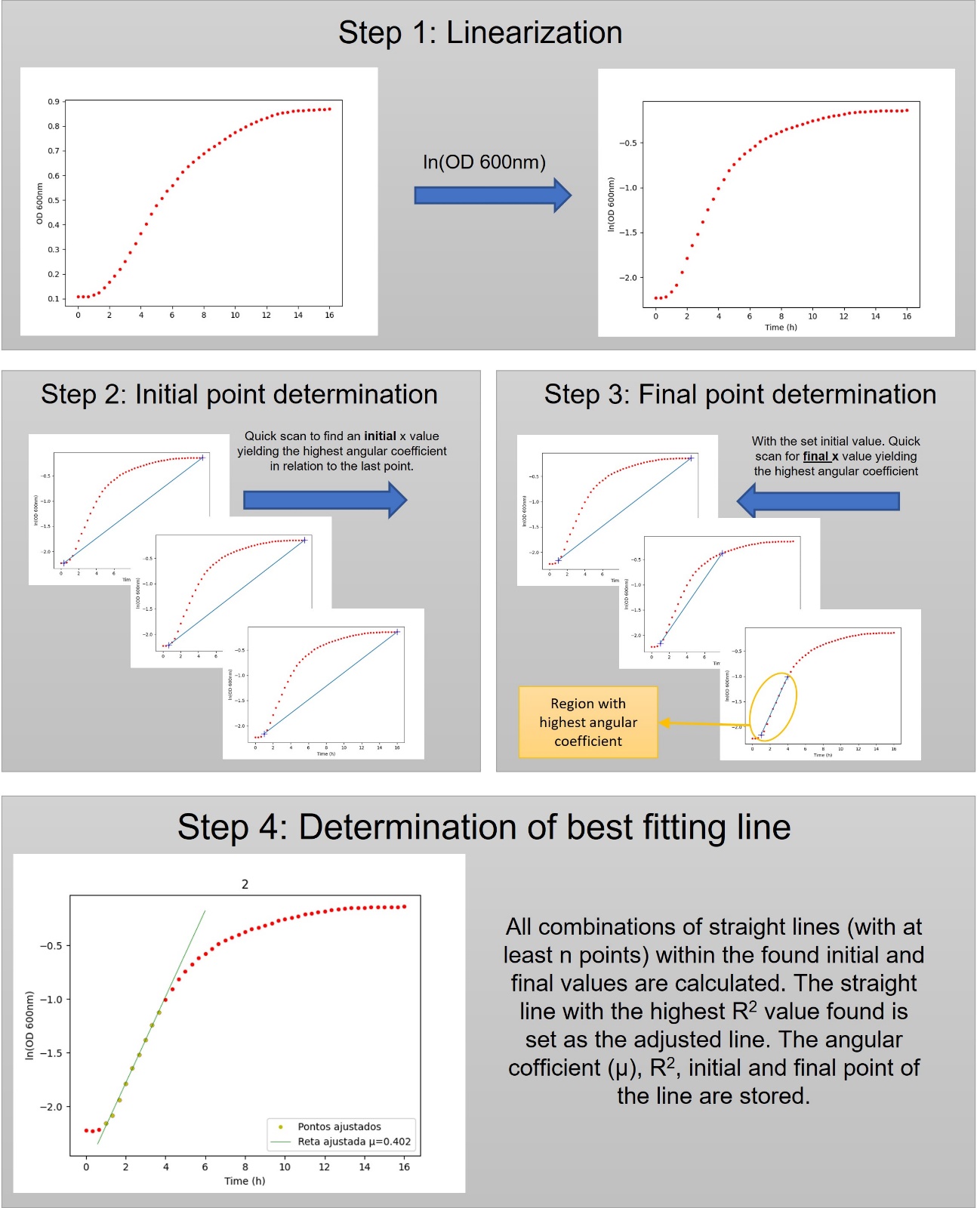


Figure S6 – Visual explanation of the algorithm used to calculate the steepest region in the ln(OD) versus time curves, and growth parameters (µ, OD_max_, and t_decel_).
